# Human fascioliasis in Argentina: retrospective overview, critical analysis and baseline for future research

**DOI:** 10.1186/1756-3305-4-104

**Published:** 2011-06-11

**Authors:** Roberto Mera y Sierra, Veronica H Agramunt, Pablo Cuervo, Santiago Mas-Coma

**Affiliations:** 1 Cátedra de Parasitología y Enfermedades Parasitarias, Facultad de Ciencias Veterinarias y Ambientales, Universidad J.A. Maza, Av. de Acceso Este - Lateral Sur 2245, S. José, Guaymallén, Mendoza, Argentina; 2 Área de Parasitología, Facultad de Ciencias Médicas, Universidad Nacional de Cuyo, Av. Libertador 80, Mendoza, Argentina; 3 Departamento de Parasitología, Facultad de Farmacia, Universidad de Valencia, Av. Vicente Andrés Estellés s/n, 46100 Burjassot, Valencia, Spain

## Abstract

In Argentina, human fascioliasis has never been adequately analysed, although having a physiography, climate, animal prevalences and lymnaeids similar to those of countries where the disease is endemic such as Bolivia, Peru and Chile. We performed a literature search identifying 58 reports accounting for 619 cases, involving 13 provinces, their majority (97.7%) from high altitudes, in central mountainous areas and Andean valleys, concentrated in Cordoba (430 cases), Catamarca (73), San Luis (29) and Mendoza (28), the remaining provinces being rarely affected. This distribution does not fit that of animal fascioliasis. Certain aspects (higher prevalence in females in a local survey, although a trend non-significant throughout Argentina) but not others (patient's age 3-95 years, mean 37.1 years) resemble human endemics in Andean countries, although the lack of intensity studies and surveys in rural areas does not allow for an adequate evaluation. Human infection occurs mainly in January-April, when higher precipitation and temperatures interact with field activities during summer holidays. A second June peak may be related to Easter holidays. The main risk factor appears to be wild watercress ingestion (214) during recreational, weekend outings or holiday activities, explaining numerous family outbreaks involving 63 people and infection far away from their homes. Diagnosis mainly relied on egg finding (288), followed by serology (82), intradermal reaction (63), surgery (43), and erratic fluke observation (6). The number of fascioliasis-hydatidosis co-infected patients (14) is outstanding. Emetine appears as the drug most used (186), replaced by triclabendazole in recent years (21). Surgery reports are numerous (27.0%). A long delay in diagnosis (average almost 3.5 years) and high lithiasis proportion suggest that many patients are frequently overlooked and pose a question mark about fascioliasis detection in the country. High seroprevalences found in recent random surveys suggest human endemic situations. This analysis highlights that human fascioliasis may have been overlooked in the past and its real epidemiological situation in high risk rural, mainly altitudinal areas, may currently be underestimated. Results provide a valuable baseline on which to design appropriate multidisciplinary studies on humans, animals and lymnaeids to assess up to which level and in which areas, human fascioliasis may represent a health problem in Argentina.

## Background

Fascioliasis, a major veterinary problem worldwide due to the economic losses it causes in animal husbandry, has recently become increasingly important in public health, with human reports increasing in number and the description of human endemic areas, comprising hypo- to hyperendemic situations in many countries of Latin America, Africa, Europe and Asia [1-4]. This emergence appears to be partly related to climate change, global warming and the so-called global change, among which mainly anthropogenic modifications of the environment and increasing short- and long-distance travel and import/export facilities available nowadays. All these phenomena have shown to have a great impact on snail-borne zoonotic diseases, as is the case of a trematodiasis very dependent on climate and environment characteristics such as fascioliasis [5-7].

The magnitude of fascioliasis impact on communities of human endemic areas, mainly on children and females [3], is due to its chronic, debilitating, and poverty-promoting characteristics, with a pathogenicity until recently considered restricted mainly to the acute phase [2,8], but which has recently proved to constitute a health problem during the very long chronic phase [9-12]. Impact and wide emergence prompted the World Health Organization (WHO) to include human fascioliasis on its list of priorities among neglected tropical diseases (NTDs) [13].

In the Americas, this helminthic disease is caused by the liver fluke *Fasciola hepatica *[4], transmitted by many different freshwater snail vectors belonging to the family Lymnaeidae, mainly species included within the *Galba/Fossaria *group [14,15]. In South America, human endemic areas have been described in Andean regions, mainly in higher altitude areas of countries such as Bolivia, Peru and Chile, and secondarily in Ecuador and Venezuela [3,16-22].

In Argentina, the situation of human fascioliasis has never been the subject of an adequate analysis. Only short reports within large worldwide reviews may be specifically acknowledged [2,8]. This is surprising when taking into account that (i) neighbouring countries such as Bolivia and Chile reported hyperendemic areas of human fascioliasis long ago [16-21], (ii) the country presents a very widely distributed veterinary problem of fascioliasis in livestock [23], (iii) it includes Andean environmental characteristics appropriate for fascioliasis transmission to humans [3,24], and (iv) recent studies have reported the discovery of lymnaeid vector species of well-known transmission capacity to humans to be the same combined haplotype of *Galba truncatula *responsible for the human hyperendemic area presenting the highest prevalences and intensities known [25,26] and *Lymnaea neotropica *[27]. Argentina is a country of high livestock production, where sheep and cattle but also equines constitute important economic sources. All these different domestic species are important reservoirs of fascioliasis and represent similar sources of infection for humans, given the results obtained in experimental studies which have demonstrated that snail-borne infective metacercarial stages originating from different animal species do not significantly differ in their infection capacity [28,29].

The purpose of the present ten-year research work is to provide an in-depth analysis of the results obtained in a thorough bibliographical search of human fascioliasis cases in Argentina. In that country, even though there are national data on animal fascioliasis, where slaughterhouse reports have been submitted to the authorities for practically a hundred years, there are, however, no official reports on human fascioliasis, because human infection by the liver fluke is not of obligatory declaration. Thus, published and unpublished written reports are the only source of information, whether they may be articles in scientific journals, books, university theses, communications at scientific meetings, or internal reports of agencies, ministries, hospitals, health centres, etc. [30].

## Review

### Description of dataset

The first case published in Argentina concerned an Arab immigrant who had just arrived in Argentina [31]. The onset of symptoms was upon arrival, indicating that the disease was most probably acquired in his homeland. The patient died and the diagnosis was made during autopsy when numerous flukes were found in the liver. Since this case was not autochthonous, it is not considered in this review.

This overview begins from the first autochthonous case diagnosed by coprology in 1924 [32]. More cases followed soon, such as a coprologically diagnosed patient from San Luis province [33], another diagnosed during surgery [34] and yet another one from San Luis province [35]. Fifty three cases had already been published prior to 1960.

The first WHO review [8] refers to only 13 human reported cases in Argentina for the 1970-1990 period, namely only those reported by Carena et al. [36]. This number of human cases was increased to 85 in the following extensive WHO initiative [2]. The present review offers a completely new picture of human fascioliasis in Argentina, including a total of 619 authochthonous cases in 58 reports of different kinds analysed up to 2010 (Table 1), which means that the number of human cases published is more than seven times greater than previously noted. Such a pronounced difference seems to be due to the great amount of overlooked local publications (and also communications at scientific meetings with abstract books of restricted dissemination). When considering that human fascioliasis infection is in Argentina of non-obligatory declaration, similarly to the rest of the world, one may conclude that the number of infected patients should be even greater than that. Interestingly, the need for Argentinian health authorities to warn about this disease was already noted long ago when fascioliasis was cited in animals in the country for the first time [37].

**Table 1:** Human fascioliasis reports in Argentina, arranged chronologically, including details on infection according to number of cases, gender, province, diagnostic method, treatment, and clinical data.

**YEAR**	**AUTHOR**	**Ref. No**.	**No. cases**	**GENDER**	**PROVINCE**	**DIAGNOSTIC METHOD**	**TREATMENT**	**CLINICAL DATA**
1924	Greenway	[32]	1	NS_(1)_	NS_(1)_	Ec_(1)_	NS_(1)_	NS_(1)_
1927	Bengolea et al.	[33]	1	F_(1)_	SL_(1)_	Ec_(1) _Es_(1)_	NS_(1)_	AP_(1)_
1928	Del Valle & Donovan	[34]	1	F_(1)_	NS_(1)_	Surg_(1)_	NS_(1)_	AP_(1) _ Nau_(1) _Ic_(1) _HA_(1)_
1930	Bacigalupo et al.	[35]	1	F_(1)_	SL_(1)_	Ec_(1) _Es_(1)_	NS_(1)_	NS_(1)_
1933	Mascheroni	[108]	1	F_(1)_	BA_(1)_	Ec_(1) _Es_(1)_	NS_(1)_	NS_(1)_
1933	Scrimaglio (In Bacigalupo et al. 1943)	[93]	1	M_(1)_	SFe_(1)_	Ec_(1) _Es_(1)_	NS_(1)_	NS_(1)_
1937	Castex & Greenway	[91]	1	M_(1)_	Cba_(1)_	Ec_(1) _Ect_(1)_	NS_(1)_	AP_(1) _Lks_(1)_
1939	Boto	[69]	1	F_(1)_	Tuc_(1)_	Es_(1)_	Em_(1)_	Eo_(1)_ AP_(1)_ Fev_(1)_ Lks_(1)_ Urt_(1)_
1939	Paladino & Galarce	[92]	1	F_(1)_	BAcity_(1)_	Surg_(1) _Ect_(1)_	NS_(1)_	AP_(1)_
1940	Cames	[76]	2	M_(2)_	Cba_(1) _SFe_(1)_	Es_(1) _Surg_(1)_	Em_(2) _MFE_(1)_	Eo_(1) _AP_(1) _Fev_(2) _WL_(1) _Ic_(1)_
1942	Bacigalupo	[52]	5_(fo1)_	F_(2) _M_(3)_	Cba_(4) _BA_(1)_	Ec_(2) _Es_(1) _CE_(2)_	Em_(4) _NS_(1)_	Eo_(4) _AP_(4) _Fev_(3) _WL_(1)_
1943	Solari & Canepa	[77]	1	F_(1)_	Cba_(1)_	Es_(1)_	Em_(1)_	AP_(1)_
1943	Bacigalupo et al.	[93]	1	M_(1)_	Cba_(1)_	Ect_(1)_	Em_(1)_	Eo_(1) _AP_(1)_
1944	Cuenya	[109]	3	F_(1) _M_(2)_	Tuc_(1) _Ju_(1) _Sal_(1)_	Ec_(3) _Es_(1)_	NS_(3)_	AP_(1)_
1947	Cid Cames et al.	[79] [80]	1	F_(1)_	Tuc_(1)_	Surg_(1) _Ect_(1)_	Em_(1)_	AP_(1) _WL_(1) _Lith_(1) _Ic_(1) _HA_(1)_
1952	Rodríguez	[38]	16	F_(3) _M_(1) _NS_(12)_	Cbsa_(15) _Cat_(1)_	Ec_(16)_	NS_(16)_	Eo_(5) _Ap_(1) _Lks_(4) _Ic_(1)_
1953	Longo & Daraio	[89]	1	F_(1)_	Sal_(1)_	Surg_(1) _NI_(1)_	Em_(1)_	AP_(1) _Urt_(1) _Nau_(1) _Lith_(1) _Vo_(1)_
1954	Petraglia	[98]	1	M_(1)_	Cha_(1)_	Ec_(1)_	Em_(1)_	AP_(1) _Eo_(1) _Fev_(1)_
1954	Rodríguez	[39]	10	F_(1) _M_(2) _NS_(7)_	Cba_(9) _Cat_(1)_	Ec_(10)_	NS_(10)_	Eo_(4) _Lks_(1)_
1955	Cáceres	[55]	1	F_(1)_	BAcity_(1)_	Surg_(1)_	Em_(1)_	AP_(1) _Fev_(1) _Ic_(1) _Lith_(1) _Vo_(1)_
1955	Logaldo	[101]	1	F_(1)_	Mza_(1)_	Surg_(1)_	Em_(1)_	Ap_(1) _Nau_(1) _Lith_(1)_
1961	Ahualli & Arias	[110]	1	F_(1)_	Tuc_(1)_	Es_(1)_	Em_(1)_	AP_(1) _WL_(1) _Nau_(1) _Vo_(1) _HA_(1) _Dia_(1)_
1961	Rodriguez	[40]	23	NS_(23)_	Cba_(22) _Cat_(1)_	Ec_(23)_	NS_(23)_	NS_(23)_
1961	"Other colleagues" cited in Rodriguez 1961	[40]	150	NS_(150)_	Cba_(150)_	NS_(150)_	NS_(150)_	NS_(150)_
1961	Strada	[72]	19	F_(1) _M_(1) _NS_(17)_	Cba_(19)_	Ec_(1) _Es_(2) _Surg_(1) _NS_(15)_	Em_(2) _NS_(17)_	Eo_(2) _AP_(3) _Fev_(3) _Lks_(1) _WL_(1) _Vo_(1) _HA_(1)_
1962	Urrutia & Ferraris	[111]	1	NS_(1)_	NS_(1)_	Surg_(1)_	NS_(1)_	NS_(1)_
1964	Cornejo & Castillo	[112]	1	M_(1)_	Sal_(1)_	Ec_(1) _Es_(1)_	Em_(1)_	Eo_(1) _Lks_(1)_
1964	Simon et al	[86]	1	M_(1)_	Mza_(1)_	Es_(1) _ID_(1)_	Em_(1)_	Eo_(1) _AP_(1) _Fev_(1) _Lks_(1) _WL_(1) _Vo_(1)_
1964	Cañas et al	[113]	1	M_(1)_	Ju _(1)_	Es_(1)_	Em_(1)_	AP_(1) _Asth_(1) _Nau_(1) _Dia_(1) _Cst_(1)_
1965	Niño	[107]	4	NS_(4)_	BAcity_(2) _NS_(2)_	Ec_(2) _Es_(2)_	NS_(4)_	NS_(4)_
1967	Sosa & Romero	[114]	2	F_(1) _M_(1)_	Cba_(2)_	Es_(2)_	Em_(2)_	Eo_(2) _AP_(1) _Fev_(2) _WL_(1) _Nau_(1) _Vo_(1) _Dia_(1)_
1967 1969	Ruggieri et al. Correa et al.	[94] [95]	1	F_(1)_	Cba_(1)_	Surg_(1) _Ect_(1)_	NS_(1)_	AP_(1) _Fev_(1) _Urt_(1) _Nau_(1) _Ic_(1) _Vo_(1) _HA_(1)_
1969	Peiretti et al.	[87]	17	NS_(17)_	Mza_(15) _SL_(2)_	Es_(5) _ID_(17)_	NS_(17)_	NS_(17)_
1969	Trossero & Nocetti	[103]	1	F_(1)_	SFe_(1)_	Surg_(1)_	Em_(1)_	AP_(1) _Fev_(1) _Ic_(1) _Vo_(1)_
1970	Padilla Antoni et al.	[99]	1	NS_(1)_	Tuc_(1)_	Surg_(1)_	NS_(1)_	Eo_(1) _AP_(1) _Vo_(1) _HA_(1)_
1972	Carena et al.	[36]	13	F_(4)_ M_(9)_	Cba_(11) _Cat_(1) _Tuc_(1)_	Es_(13)_	Em_(13)_	Eo_(13) _AP_(8) _Fev_(1) _Asth_(3) _Urt_(2) _HA_(2) _Cst_(1) _Dia_(1)_
1972	Ossola et al.	[49]	12_(fo3)_	F_(7) _M_(5)_	Cba_(12)_	Ec_(2) _Es_(2) _ID_(12)_	NS_(12)_	Eo_(11) _AP_(12) _Fev_(9) _WL_(8) _Asth_(10) _Urt_(4) _Ic_(3) _Cst_(3) _Dia_(2)_
1973	Sonzini Astudillo et al.	[104]	5	F_(4) _M_(1)_	NS_(5)_	Es_(1) _Surg_(4)_	Em_(1) _NS_(4)_	Eo_(1) _AP_(4) _Asth_(1) _Lith_(3) _Ic_(4) _HA_(1)_
1973	Peiretti & Morales	[50]	4_(fo1)_	F_(1) _M_(3)_	SL_(4)_	ID_(4)_	Em_(4)_	Eo_(4) _AP_(4) _Fev_(2) _Lks_(2) _WL_(1) _Ic_(1) _Cst_(1) _Vo_(1)_
1981	Majul et al.	[102]	6	NS_(6)_	NS _(6)_	Es_(2) _Surg_(6)_	Em_(6)_	Eo_(2) _AP_(6) _Lith_(6)_
1981	Alaggia (in Andrada et al., 1983 [90])	[100]	16	NS_(16)_	NS_(16)_	Surg_(16)_	NS_(16)_	NS_(16)_
1982	Pizzi et al.	[106]	54	NS_(54)_	Cba_(54)_	Ec_(54)_	NS_(54)_	NS_(54)_
1982	Siciliano	[41]	101	F_(50) _M_(51)_	Cba_(101)_	Ec _(61) _Es_(16) _ID_(29)_	Em_(97) _Clq_(4)_	Eo_(87) _AP_(78) _Fev_(58) _Lks_(69) _WL_(56) _Anrx_(53) _Asth_(67) _Urt_(37) _Nau_(31)_
1983	Andrada et al.	[90]	5	F_(5)_	Cat_(5)_	Surg_(4) _NI_(5)_	Em_(5)_	Eo_(3) _AP_(5) _Urt_(1) _Ic_(2) _Lith_(2)_
1983	Giffoniello et al.	[78]	2	F_(1) _M_(1)_	Cba_(2)_	Es_(1) _Surg_(1) _NI_(1)_	Em_(2)_	Eo_(2) _AP_(2) _Fev_(2) _WL_(1) _Asth_(1) _Lith_(1) _Vo_(1) _HA_(1)_
1985	Miguel et al.	[97]	5	NS_(5)_	BA_(3)_ Mza_(1)_ For_(1)_	Es_(5)_	Em_(5)_	Eo_(1)_ AP_(5)_ Lks_(4)_ Ic_(2)_
1989	Siciliano et al	[42]	15(fo2)	F_(9)_ M_(5)_ NS(1)	Cba_(15)_	Ec_(12) _Es_(1)_ Surg_(2)_	Em_(15)_	Eo_(14)_ AP_(15)_ Fev_(15)_ Lks_(10)_ Urt_(8)_ Ic_(2)_
1991	Melero et al.	[88]	1	M_(1)_	SL_(1)_	Es_(1)_ NI_(1)_	Tcl_(1)_	Eo_(1)_ AP_(1)_ Fev_(1)_ Lks_(1)_ WL_(1)_
1995	Minoprio et al.	[51]	5(fo1)	F_(3)_ M_(2)_	Mza_(5)_	Ect_(1)_ CE_(4)_ NI_(3)_	Tcl_(5)_ Pzq_(1)_	Eo_(3)_ AP_(2)_ Fev_(2)_ Lks_(1)_ Asth_(2)_ Urt_(3)_ Ic_(1)_
2000	Ale et al.	[115]	1	F_(1)_	SL_(1)_	Ec_(1)_	NS_(1)_	Eo_(1)_ Lks_(1)_
2005	Carnevale (in Rubel et al. 2005)	[61]	4	NS_(4)_	SL_(4)_	Ser_(4)_	NS_(4)_	NS_(4)_
2005	Rubel et al.	[61]	1	F_(1)_	Neu_(1)_	Ser_(1)_	Tcl_(1)_	Eo_(1)_ AP_(1)_ Fev_(1)_ Lks_(1)_
2005 2006	Lloret et al. Salomon et al.	[54] [48]	5(fo1)	M_(5)_	Mza_(5)_	Ec_(2) _Es_(1)_ Ser_(2)_ CE_(2)_	Tcl_(5)_	Eo_(5)_ AP_(5)_ Lks_(5) _WL_(2)_ Fev_(5) _Dia_(2)_ Urt_(3)_
2006	Corti et al.	[85]	1	F_(1)_	Cba_(1)_	Ser_(1)_ NI_(1)_	Tcl_(1)_	Eo_(1)_ Fev_(1)_
2008	Rios et al.	[62]	1	M_(1)_	Neu_(1)_	Ec_(1)_ Ser_(1)_	Alb_(1)_	Eo_(1)_ AP_(1)_ Fev_(1)_ Lks_(1)_ Asth_(1)_ Urt_(1)_
2008	Nieto Sosa et al.	[47]	23(fo2)	NS_(23)_	Cba_(8)_ SL_(15)_	Ec_(23)_ Ser_(9)_	Em_(15)_ Tcl_(8)_	Eo_(23)_ AP_(23)_ Fev_(23)_ Lks_(23)_
2009	Malandrini et al.	[43]	54	F_(37)_ M_(17)_	Cat_(54)_	Ser_(54)_	NS_(54)_	NS_(54)_
2009	Malandrini et al.	[53]	10	NS_(10)_	Cat_(10)_	Ser_(10)_	NS_(10)_	NS_(10)_

Abbreviations: **F**: female; **M**: male; **NS**: not specified; **Fo**: family/group outbreaks.

Provinces: **BA**: Buenos Aires; **BAcity**: Buenos Aires city; **Cat**: Catamarca; **Cha**: Chaco; **Cba**: Córdoba; **For**: Formosa; **Ju**: Jujuy; **Mza**: Mendoza; **Neu**: Neuquén; **Sal**: Salta; **SL: **San Luís; **SFe**: Santa Fé; **Tuc**: Tucumán.

Treatment: **Em**: emetine; **Tcl**: triclabendazole; **Alb**: albendazole; **MFE**: male fern extract; **Pzq**: praziquantel; **Clq**: Cloroquine.

Diagnostic Method: **Ec**: egg observation in coprological sample; **Es**: egg observation in sample obtained by sondage; **ID**: intradermal test; **Surg**: surgical; **CE**: clinical/epidemiological; **Ect**: ectopic presentation; **NI**: non invasive image-based diagnosis; **Ser**: serology.

Clinical Data: **AP**: abdominal pain; **Anrx**: anorexia; **Asth**: asthenia; **Cst**: Constipation; **Dia**: diarrhea; **Eo**: eosinophilia; **Fev**: fever; **HA**: headache; **Ic**: ictericia; **Lks**: leucocitosis; **Lith**: lithiasis; **Nau**: nausea; **Urt: **urticaria; **Vo**: vomiting; **WL**: weight loss.

Note: number of cases noted in parenthesis

**Figure 1:**
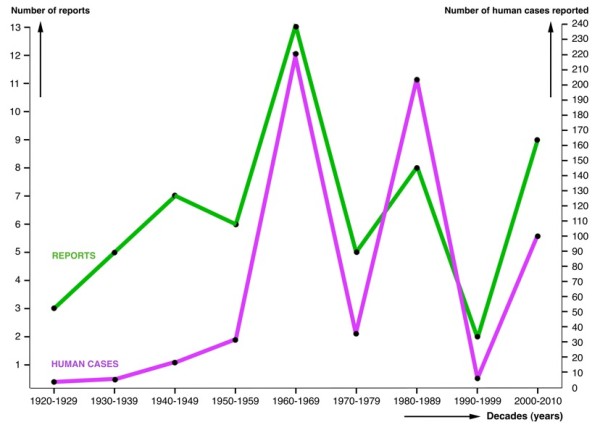
**Reports on human fascioliasis in Argentina**. Evolution of the number of reports on human fascioliasis and number of human infection cases in Argentina according to decades.

Both report and case numbers follow a parallel evolution with quite important fluctuations (Figure 1). This result is most likely to be linked to particular circumstances encouraging physicians to publish their diagnosed cases rather than a real reflection of the annual evolution of the epidemiological situation. For instance, only two authors, C. Rodriguez and C. Siciliano [38-42], are responsible for 51% of the cases reported in Argentina, the decades when they were active publishing appearing as those decades with the greatest amount of cases (1960's and 1980's).

### Characteristics of the infected population

In only 267 cases did the authors specify the gender of the patients: 120 (44.94%) were male and 147 (55.06%) were female. A somewhat higher preference for females (68.52% out of 54 subjects) was also found in the serological study performed with ELISA by Malandrini and collaborators [43] in the locality of Taton, Tinogasta, Catamarca, the only randomized survey carried out in Argentina so far. Unfortunately, no studies on eggs per gram of faeces (epg) have been perfomed in Argentinian patients, so that a gender relationship with intensity could not be assessed. Although that apparent preference for females is not statistically significant in Argentina, this trend is consistent with what has been described in human endemic areas: significantly higher intensities in females in Bolivia and Peru [19-22], and significantly higher prevalences in Chile [17] and Egypt [44]. However, such a female preference rule does not always appear to be significant at prevalence level [19-22,45,46].

**Figure 2:**
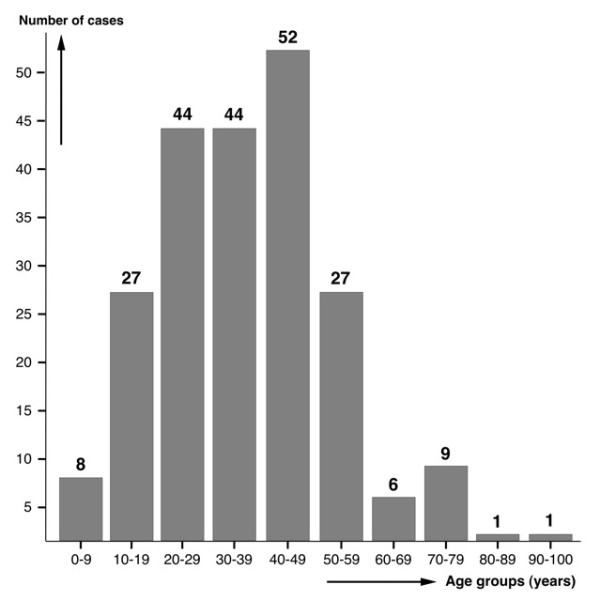
**Age of fascioliasis patients**. Age distribution in 219 patients in whom fascioliasis was diagnosed.

Age in years was specified in 219 (35.38%) patients and noted for guidance only (e.g., child, adult) in another 12. The range was from 3 [41] to 95 years of age [43] (average 37.09 ± 17.07 years) (Figure 2). In Argentina, the only random survey detected positivity from small children to old individuals, without age preference [43]. This absence of age correlation contrasts with other countries, where prevalences and intensities peak in the 9-11 age group, although adults and old age groups may also show high infection rates, as is the case of Bolivia [19-21], Peru [22] and Egypt [44]. However, no differences between age groups were found in Chile [17] or Iran [45].

In Argentina, several outbreaks presenting typical foodborne characteristics appear related to the most common risk factor: ingestion of watercress naturally growing along the river- and stream-beds picked during recreational, weekend or vacation activities. Many of these field excursions are undertaken by a family or as a group activity. This explains why family outbreaks have been noted to be common, whereas isolated cases seem to be rare in the country [47]. Outbreaks described in eleven families involving a total of 63 people [42,47-52], including a maximum of up to 15 family members affected at once [47], are good examples. However, results obtained in a recent serological survey of a local resident population in Catamarca [43] show that not all situations including several infected subjects are in fact family outbreaks.

### Biogeographical aspects

#### Geographical distribution according to provinces

The geographical origin of the patients was specified in 587 patients (94.83%), whereas in 32 cases (5.17%) not even the province of origin was noted. Sometimes only the province where the infection occurred was mentioned, in other instances the specific locality was also added.

**Figure 3:**
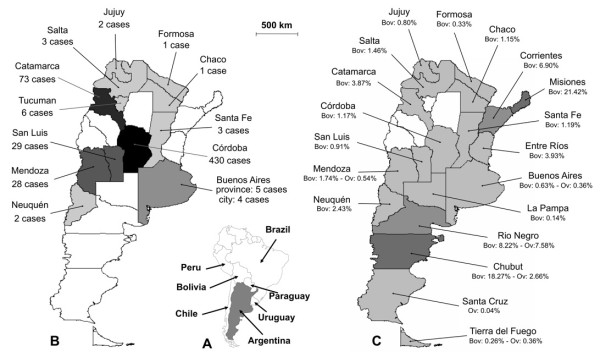
**Geographical distribution of fascioliasis in Argentina**. A) country location in the southern cone of South America; B) distribution of human fascioliasis infection reports (number of cases), according to provinces where infection was presumed to have occurred; C) distribution of fascioliasis in livestock including province prevalences according to slaughterhouse condemnation data for the 2006-2009 period provided by the Servicio Nacional de Sanidad y Calidad Agroalimentaria (SENASA) (bov = bovines; ov = ovines).

Human cases have been found in 13 out of the total of 23 provinces plus Buenos Aires capital, covering more than half (51.48%) of the total surface of continental Argentina. Cordoba, Catamarca, San Luis and Mendoza include the largest number of patients detected, the remaining provinces being only rarely affected (Table 2 Figure 3). Human infection has most probably been completely overlooked in La Rioja and San Juan provinces, with no case reported despite being surrounded by the aforementioned provinces. Adequate studies are needed to assess whether human infection occurs in La Rioja and San Juan given their physiographic and climatic characteristics which suggest infection risks similar to those in the aforementioned neighbouring ones.

**Table 2:** Evolution of human fascioliasis infection reports in Argentina, according to provinces where infection was presumed to have occurred, number of cases and respective articles furnishing the information

**Province**	**Total No. of cases**	**No. of cases per individual references** **(ordered according to year of publication)**	Observations
Cordoba	430	1 case published in 1937 [91]; 1 in 1940 [76]; 4 in 1942 [52]; 1 in 1943 [93]; 1 in 1943 [77]; 1 in 1944 [109]; 5 in 1952 [38]; 9 in 1954 [39]; 22 in 1961 [40]; 150 in 1961 [unpublished by other colleagues, cited in 40]; 19 in 1961 [72]; 2 in 1967 [114]; 1 in 1967 & 1969 [94,95]; 11 in 1972 [36]; 12 in 1972 [49]; 54 in 1982 [106]; 101 in 1982 [41]; 2 in 1983 [78]; 15 in 1989 [42]; 1 in 2006 [85]; 8 in 2008 [47]	Two papers deal with the same case [94,95]
Catamarca	73	1 case published in 1952 [38]; 1 in 1954 [39]; 1 in 1961 [40]; 1 in 1972 [36]; 5 in 1983 [90]; 54 in 2009 [43]; 10 in 2009 [53]	these 73 cases do not include 10 serologically suspicious patients who could not be confirmed due to absence of eggs in stools [116]
San Luis	29	1 case published in 1927 [33]; 1 in 1930 [35]; 2 in 1969 [87]; 4 in 1973 [50]; 1 in 1991 [88]; 1 in 2000 [115]; 4 in 2005 [Carnevale in 61]; 15 in 2008 [47]	these 29 cases include an 11% seropositivity found in 34 samples obtained randomly in the population by Carnevale [unpublished data in 61]
Mendoza	28	1 case published in 1955 [101]; 1 in 1964 [86]; 15 in 1969 [87]; 1 in 1985 [97]; 5 in 1995 [51]; 5 in 2005 & 2006 [48,54]	Two papers describe the same outbreak [48,54]
Tucuman	6	1 case published in 1939 [69]; 1 in 1944 [109]; 1 in 1947 [79,80]; 1 in 1961 [110]; 1 in 1970 [99]; 1 in 1972 [36]	Two papers refer to the same case [79,80]
Buenos Aires	5	1 case published in 1933 [108]; 1 in 1942 [52]; 3 in 1985 [97]	
City of Buenos Aires	4	1 case published in 1939 [92]; 1 in 1955 [55]; 2 in 1965 [107]	
Salta	3	1 case published in 1944 [109]; 1 in 1953 [89]; 1 in 1964 [112]	
Santa Fe	3	1 case published in 1940 [76]; 1 in 1933 [Scrimaglio in 93]; 1 in 1969 [103]	
Neuquen	2	1 case published in 2005 [61]; 1 in 2008 [62]	
Jujuy	2	1 case published in 1944 [109]; 1 in 1964 [113]	
Chaco	1	1 case published in 1954 [98]	
Formosa	1	1 case published in 1985 [97]	
Province not specified	32	1 case published in 1924 [32]; 1 in 1928 [34]; 1 in 1962 [111]; 2 in 1965 [107]; 5 in 1973 [104]; 16 in 1981 [100]; 6 in 1981 [102]	
Total: 13 provinces	619 cases	58 reports	

The pronounced concentration of cases in the Cordoba and Andean areas is worth mentioning, although not in absolute numbers, at least with regard to the proportion of human case distribution. Unfortunately, human community surveys (active detection) have not been undertaken. All reports concern symptomatological subjects who voluntarily seek medical assistance (passive detection), except the only survey performed [43,53] whose results increased the human case number for Catamarca province from 9 to 73.

**Figure 4:**
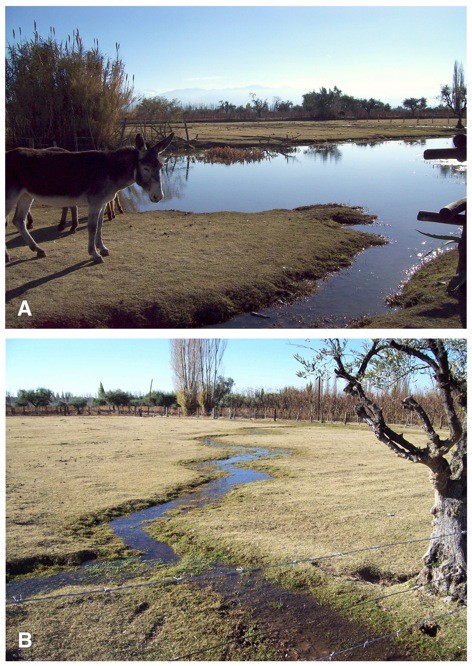
**Human infection risk**. A recreational farm in the locality of Perdriel (902 m altitude), Mendoza province, well known because of its great touristic attraction, mainly during holiday periods and weekend outings: A) water collections of an artifical pond, and B) the overflow originated from an artificial irrigation channel, where the snail vector species *Lymnaea neotropica *was collected and cattle, goats, horses, donkeys and llamas proved to be infected [27].

The very high case number in Cordoba results from patients diagnosed by different physicians throughout many decades (see Table 2), mainly at the end of the 50s and beginning of the 60s as noted in the compilation, made by one active Cordobese author, of the many patients diagnosed by himself plus the 150 patients diagnosed by other Cordobese colleagues, and which he presented at the Primer Congreso Médico Sanitario de la Provincia de Cordoba, held in La Falda on August 1957 [see [40]. Although of course such a pronounced case number difference when comparing Cordoba with other provinces may in part be the consequence of a bias due to the absence of similarly actively publishing authors in the other provinces, the very high case number in Cordoba merits an analysis. The literature review suggests an explanation related to the very large number of villages and towns playing an important role in recreational, weekend or holiday activities. These recreational areas attended by thousands of tourists, campers or weekend visitors overlap with areas in which lymnaeids and animal fascioliasis are present (Figure 4) [27]. In such places, the infection risk for a large amount of people is greater than in scarcely inhabited Andean areas. Moreover, the social, cultural and economic level of people spending holidays in such areas is high and the likelihood for them to seek appropriate medical assistance and obtain a correct diagnosis is greater than for people living in rural areas.

This highlights that people may become infected in a place different, sometimes even far or very far away, from the place where they live. Several family outbreaks described in Cordoba province support this assumption: the first such outbreak in La Calera [52], another family infected during a picnic, another during a few days camping, another family buying uninspected watercress sold by a street vendor [49], and finally an eight-member family in La Punilla [47]. In San Luis province, an outbreak involved two families that camped together in El Volcan [50], and another affected 15 family members in Merlo [47]. Similar family outbreaks occurred in Mendoza province, one involving five members [51] and another with five members infected during a trip to the Andean region of San Carlos [48,54].

#### Distribution according to altitude

Even though human infection risk is present in many geographical regions of the country, data indicate higher probabilities in given high altitude areas. Indeed, the great majority of cases including information on infection place (574: 97.79%), are from hilly or mountainous areas. Human reports appear concentrated in: (i) the central mountainous areas of Cordoba and San Luis, and (ii) the Andes mountains, mainly in Andean valleys.

Cordoba, the province with the greatest amount of cases, is a good example, with practically all human cases coming from its western mountainous areas (Sierras de Cordoba), despite higher precipitation rates and livestock abundance in its eastern lowland plains. Nevertheless, a possible sample bias cannot be ruled out in this case concentration due to the extensive patient-record publication by two local authors [38-42]. Similar situations are found in San Luis and Mendoza provinces, both with all human cases from their western mountainous areas, instead from their eastern plains.

Only 13 cases (2.21%) originated from areas near sea level and flat terrain, namely from Buenos Aires province and City, Sante Fe, Chaco and Formosa. These few reports were all related to important rivers. One interesting case was a patient from Buenos Aires city who declared not having left the city in the previous 17 years [55].

Such a case concentration in mountainous areas is not unlike Bolivia, Peru and Chile, where human endemic areas are linked to altitude areas, as a consequence of both (i) geographical distribution of the main lymnaeid vectors involved in transmission to humans restricted to or preferring such altitude areas [56], and (ii) the greater liver fluke transmission capacity in high altitude areas [24]. This suggests the appropriateness of verifying whether human fascioliasis endemic situations may also exist in high altitude areas of Argentina.

#### Links to livestock infection

The distribution of human infection does not appear to fit the one of animal fascioliasis, which covers the whole country according to official slaughterhouse records (Figure 3). In this respect certain areas are worthy of note, such as Corrientes province with absolutely no human case reported in the literature but high prevalences in livestock [57-60], and Neuquen province with only two human cases reported [61,62] despite a very high prevalence in cattle [63].

A similar lack of geographical fit between human and animal fascioliasis has already been seen in other countries [3,4]. Unfortunately, in Argentina it becomes impossible to ascertain whether this is the real situation or only a distorted picture due to incomplete data. Lack of appropriate human community surveys in areas with high prevalences in animals, absence of human-case reporting due to non-obligatory declaration, and overlooked human infection related to misdiagnosis or to inhabitants of rural areas not attending health centres for diagnosis may explain such situations.

However, the lack of geographical fit in question may also be due to an altitude factor, in its turn related to both (i) altitudinal selection of lymnaeid vector species with ecological characteristics adequate for transmission to humans and (ii) altitudinal climatic factors enhancing *F. hepatica *life cycle development, as already demonstrated in other Andean countries [24,56]. Concentration of human cases in hilly or mountainous areas support this altitude explanation.

#### Seasonality

Both fasciolid life cycle and lymnaeid population dynamics are markedly dependent on climate, mainly temperature and rainfall [5-7,64]. This climatic influence is evidenced by three different transmission patterns which define human and animal infection characteristics [3,65]: monoseasonal, biseasonal and annual, permanent depending on the existence of one rainfall concentration period per year, two of them, or appropriate water body availability throughout the year [5,56]. Sometimes seasonality is related to the ingestion of contaminated plants, with most human cases occurring during the watercress season [2].

To estimate the moment when metacercariae were ingested, a prepatent period of 2-4 months should be considered. This overlaps with the acute phase, egg appearance in faeces marking the beginning of the chronic phase [66]. For instance, in Europe, human infection takes place in summer and autumn and symptoms appear in winter, and a prolonged and wet summer has often been followed by an outbreak [2].

In Argentina, a total of 110 case reports were found in which the month of the first appearance of symptoms was noted. Most of them (97) corresponded to reports from Cordoba province, of which 93 had already been analysed from that point of view [41].

**Figure 5:**
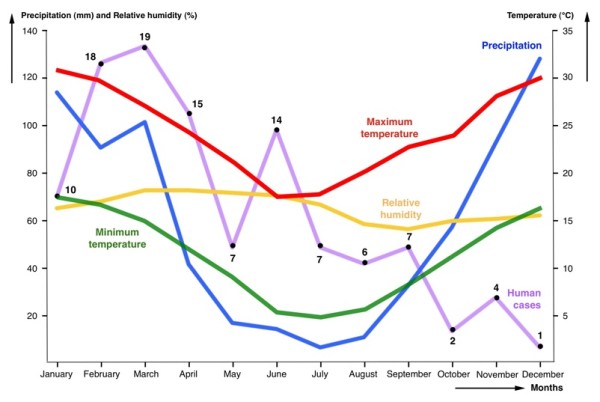
**Human fascioliasis monthly incidence**. Distribution of human fascioliasis incidence according to the month in which symptoms appear, compared with the annual distributions of mean monthly data of precipitation, humidity, and maximum and minimum temperatures. Data concerns the province of Cordoba in the 1960's and 1970's when the patients were infected.

A first pronounced January-April peak appears in the monthly distribution of these 110 cases. When comparing case distribution with the annual distributions of mean monthly data of precipitation and humidity and with mean monthly data of maximum and minimum temperatures for Cordoba province concerning the decades 1961-1970 and 1971-1980 during which the patients were infected (Figure 5), significant correlations with monthly precipitation, monthy maximum temperature and monthly minimum temperature appeared when time lags of 2 months (p values of 0.013, 0.008 and 0.0069, respectively), 3 months (p values of 0.003, 0.0049 and 0.0035, respectively) and 4 months (p values of 0.0389, 0.0293 and 0.0358, respectively) were considered. This fits with the logical delay between infection moment and symptom appearance and diagnosis. However, this largest peak of January-April may not only be due to higher precipitation and temperature data. Many people enjoy summer holidays in January and February in Cordoba province, so that the increase of recreational field activities may also interact with rainfall and temperature increases in inducing this monthly January-April incidence peak.

There is a second peak in June which poses a question mark, as no such climatic correlation appears. Other factors may be involved, such as the second yearly increase of recreational field activities during the Easter holidays. Metacercariae are known to keep their infectivity for months (even up to two years) under adequate environmental conditions [28], so that remaining metacercariae, namely those already available but not having been ingested from January, may be the cause of that delayed incidence peak.

Human infection mainly during the first months of the year is also suggested in another 15 case reports from Cordoba, in which the first month with symptoms was unfortunately not specified (therefore not included in the aforementioned statistical analysis) but the season of autumn (i.e., 21 March to 21 June) was noted [42].

However, in Argentina other transmission patterns and human infection risk seasonalities/periodicities may not be ruled out according to the large physiographical heterogeneity of the different endemic areas of such a vast country.

#### Relationships with annual climate changes

The climatic dependence of fascioliasis is also known to modify the interannual distribution of human case detection, with increases in years with heavy rainfall [2,66].

**Figure 6:**
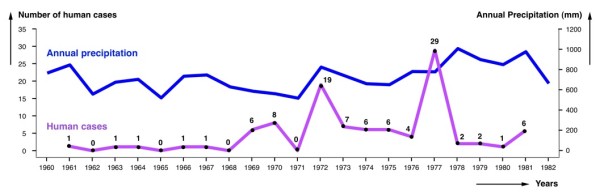
**Human fascioliasis annual incidence**. Distribution of human fascioliasis incidence according to the year in which symptoms appear, compared with annual precipitation data. Data concerns the province of Cordoba in the 1960-1982 period during which the patients were infected.

When analysing the evolution of the human case number in the whole country according to decades, one peak appears in the 1960s and another one in the 1980s (Figure 1). The annual human case number for a more detailed study was, however, only sufficient when restricted to Cordoba province during the 1960's and 1970's. The comparative analysis with annual precipitation (Figure 6) shows that the 1972 outbreak may have been caused by the sudden increase of rainfall in that year in Cordoba province, as already highlighted a long time ago [41]. However, we could not find any significant correlation.

The most pronounced outbreak in 1977 does not appear to have any apparent climatic causal origin, although it was argued that this year was the one with the largest amount of precipitation during the 1973-1981 period [41]. We have confirmed that the rainiest year in the Cordoba area in question was 1978 and not 1977. Thus, this second human report increase may have been linked to a passing fad or temporary professional trend for reporting.

#### Sources of human infection

Ingestion of freshwater plants carrying attached infective metacercariae, among which preferentially watercress, is known to constitute the main fascioliasis infection source in humans worldwide [3,67]. The consumption of aquatic plants other than watercress, as described in the hyperendemic area of Bolivia [30], is not a common practice in Argentina. Watercress is almost the only aquatic wild plant regularly consumed. In this country, with the highest beef consumption in the world, it is traditional to accompany barbecue meat ("asado") with salad. When camping or doing outdoor activities, it is very common to go to a stream and collect watercress to cover for the absence of salads.

Previous watercress ingestion has been described in 214 patients. A relationship between watercress and fascioliasis was described already long ago [33,35] and has recently been highlighted again in an editorial article [68]. Among a total of 101 cases reported from Cordoba, 94 (93,07%) had a history of watercress consumption [41].

Besides watercress, there are a very few reports which relate fascioliasis to other plants. In two cases from Cordoba province, ingestion of dandelion (*Taraxacum officinale*) was noted as a possible infection source [41]. Two other patients are mentioned to have chewed on grass that grew on a riverbank [69].

Water consumption has also been involved as a human infection source [58]. In Argentina, natural water was already presumed to be an infection source for livestock as early as two centuries ago [37]. With regard to humans, contaminated water as a possible infection source was noted in only two patients who mentioned having drunk natural water from mountain streams [51,69].

### Diagnostic aspects

#### Methods and techniques used

The diagnosis of human fascioliasis poses well-known methodological and technical problems [3,70,71]. In Argentina, the diagnostic method used was specified in the majority of the human patient reports analysed (454 cases: 73.34%) (Table 1). In several cases, even more than one method/technique was used. The diagnostic method was not specified when referring to 150 unpublished cases detected by other colleagues [40], and it was insufficiently clarified in 15 other cases mentioned in an endnote as "patients having been diagnosed while in press" [72].

Fascioliasis diagnosis in Argentina has usually relied on traditional techniques, mainly egg detection (288 positive patients), followed by common serological techniques (82), intradermal reaction (63), fluke finding during surgery (45), and erratic fluke observation (6). Sensitivity and specificity of these techniques are far from the standards in more modern ones [46,73,74]. Additionally, the absence of the use of quantitative methods, such as the standardized Kato-Katz technique to assess both pathogenicity and intensity for adequate treatment dose selection [75], is worth mentioning.

An historical account (see Table 1) shows that diagnosis by egg identification was the method used from the very beginning and, although still used in the last decade, surgical findings were common during the 1969-1989 period and serology from 2005 onwards. The intradermal test appears to have been used only during the 60's and at the beginning of the 70's and abandoned after 1982. Other methods based on clinical/epidemiological observations, non-invasive techniques, and ectopic presentations have been only sporadically applied.

The long delay with which many patients were diagnosed should be emphasized. In given reports on 26 patients, the time elapsed between the appearance of symptoms and confirmation of infection by appropriate diagnosis is noted in days or sometimes years. Calculated delay average is very high, of 1262 days, nearly three and a half years, and there are references about patients having suffered from symptoms for ten or more years without diagnosis [33,34,76-80]. This suggests either infected subjects not looking for professional diagnosis due to mild symptoms of low fluke burdens and/or misdiagnosis of patients due to the non-pathognomonic clinical picture, easily confused with other diseases when the patient attends a health centre not used to dealing with fascioliasis [81].

#### Egg finding

Coprological analyses were performed in 278 patients, including positive egg finding in 221 (79.50%). Details about the coprological technique used were very rarely described. Where noted, the Charles Barthelemy sedimentation technique, M.I.F., flotation, Baerman and multiple concentrations were mentioned, although without giving further specifications [40]. Other techniques, such as the veterinary Lumbreras Rapid Sedimentation technique [42] and those of Faust and Sheather [48], have been also used in Argentina.

Duodenal sondage was performed in 90 patients, yielding egg detection in 67 (74.44%). Interestingly, nine cases appeared negative in a coprological examination, while positive in duodenal sondage.

Despite egg finding still being the gold standard today, problematic situations giving rise to overlooking an infection may be taken into account, such as (i) absence of egg shedding during the acute phase, (ii) no egg production by given fluke strains when in humans, and (iii) lack of sufficient sensitivity in light infections. These are common in human sporadic infections in animal endemic areas, as appears to be the case in many reports from Argentina, e.g. in travellers, weekend family outings, and tourists. Thus, the widespread use of an egg finding technique as the single method for patient diagnosis may have been the reason for overlooking human infection in Argentina in the past.

Unfortunately, no study on egg size variability in human stools has been performed in the country. Consequently, given the absence of *F. gigantica*, the recently corrected egg size ranges to be henceforth used in Argentina are 100.6-162.2/65.9-104.6 μm in humans and 73.8-156.8/58.1-98.1 μm in animals [82], which are pronouncedly different from the range of 130-150/63-90 μm previously used both for humans and animals worldwide.

#### Serology

Its use in Argentina has been reported only recently, despite serological techniques having been available for this disease for a long time [71]. However, an ELISA developed locally by means of recombinant procathepsin L cystein proteinase was successfully assayed in test serum samples from 16 coprologically positive patients [83], and a Micro-ELISA method showed a sensitivity of 100% and a specificity of 97% when applied to 22 test serum samples from patients with fascioliasis infection previously verified by coprology, surgical observation, or retrograde cholangiopancreatography, thus proving to be highly useful, mainly for the previous screening of a large amount of samples [84]. The technique specified was such an ELISA in 69 cases [43,48,53,61,62]. In another case, an ELISA without further details specified was employed [85].

In 13 patients the serological technique applied was not mentioned [Carnevale, unpublished data in 47,62]. In three patients diagnosed by coprology or duodenal sondage, serology appeared positive, being negative in two other patients in whom eggs were not found [48]. Surprisingly, when applying serology to 11 patients shedding eggs in their stools, only nine of them gave a positive serological result [47]. The two coprologically positive although serologically negative patients suggest either spurious cases (fluke eggs in transit after infected livestock liver ingestion) or lack of sufficient sensitivity of the serological test applied. The opposite, that is negative coprology and positive serology, was found in another case [85]. In many human case reports, serology was the sole diagnostic method used [43,53,61].

Serological diagnosis was mentioned to have been used in the only article dealing with a random human survey performed in Argentina up to the present. In 148 randomly selected subjects from Tinogasta, province of Catamarca, ELISA was positive in 54 of whom (36.48%), as well as in ten among other 14 patients from the same locality [43,53].

#### Intradermal tests

This diagnostic method, today known to be insufficiently specific [70] and considered obsolete for the diagnosis of individual patients although still potentially useful as a quick indicator within broad field screening, seems to have been quite commonly used in Argentina in the sixties, seventies and eighties (in chronological order): 1 case [86], 17 cases [87], 12 cases [49], 4 cases [50], and 29 cases [41]. Since there has never been a commercial or standardized test, details on antigen and correlation with other diagnostic tests noted in the aforementioned articles need to be taken into account.

#### Diagnosis by non-invasive techniques

Many image-based diagnostic techniques are useful for fascioliasis [2,8,70] and have also been applied in Argentina since the eighties, although mainly for initial detection followed by confirmation by another more specific diagnostic technique [51,78,85,88-90].

#### Ectopic cases

In a 25-year-old male patient, a fluke was eliminated through the urethra [91]. In a female patient undergoing surgery for appendicitis, a fluke was removed from the appendix [92]. A *F. hepatica *specimen was found when surgically opening a tumor at the level of the last rib in a 32-year-old man who lived in the endemic zones of Cordoba, San Luis and Mendoza [93]. In a 35-year-old woman from Tucuman, *F. hepatica *eggs were found in peritoneal granulomas adhered to the gall bladder and near to the transverse colon [79,80]. Another case of intracranial fascioliasis was reported in a 44-year-old female from Cordoba [94,95]. More recently, a case of cutaneous fascioliasis was described in a 26-year-old male from Mendoza [51].

#### Clinical/epidemiological diagnosis

In some cases, patients were diagnosed based on clinical and epidemiological characteristics compatible with fascioliasis even though coprological or serological analyses yielded negative results.

In an outbreak, four family members living in the same dwelling had shared meals including watercress and presented compatible symptoms, although eggs were only found in two of them [52,93]. One of the coprologically negative patients had eosinophilia, fever and generalized pain. Symptoms disappeared after emetine treatment and in two months the patient had gained 7 kg weight. The other negative patient also showed eosinophilia (52%) and suffered generalized pain but no temperature. After treatment, pain disappeared and eosinophilia diminished to 14%.

In another family outbreak [51], the wife of a male patient showing a cutaneous fluke, had pruritic skin lesions, eosinophilia and compatible images in the gall bladder during ecography later confirmed by CATScan; her symptoms disappeared after triclabendazol treatment. The mother had asthenia, urticaria, and multiple gallstones that did not allow for the visualization of parasites through ecography; her symptoms also subsided after treatment. The brother-in-law had eosinophilia and fever, and became asymptomatic after triclabendazole selftreatment. A fifth patient, a 14-year-old girl who participated in the same family outing, had hepatomegalia and urticaria, compatible images upon ecography, and also recovered after triclabenzadole medication.

After consuming wild watercress during an outing, five persons showed compatible symptoms, including eosinophilia and leucocytosis. In three of them diagnosis was confirmed by coprology and/or serology. In the remaining two patients, analyses yielded negative results but they were considered as having been infected when recovering after fascioliasis treatment [48].

### Medical aspects

#### Clinical findings

Symptoms, laboratory results and their frequency do not appear to differ from those described elsewhere. Articles reviewed are very diverse in nature. Some include detailed clinical descriptions, but many are merely an enumeration of human cases with very vague or sometimes even no accompanying clinical data at all. Thus, information has to be treated with caution. Establishing prevalences of symptoms in the total population of patients becomes impossible, and assumptions may only be obtained from the few articles in which the symptomatology was sufficiently described (Table 1).

Of 225 patients in whom eosinophil counts where performed, 198 (88.00%) had eosinophilia. In 143 patients, authors did not note the eosinophil level. Among those in whom it was quantified, the mean was 28.00% (± 21.33%). Very high counts were found in some patients, with a maximum of 84% [69].

Leucocytosis was found in 128 of 167 patients (76.65%) in whom leucocyte counts were analysed, with a mean of 12478.72 (± 7917.89), the lowest value of 4,400, and the maximum of 52,600 found in the aforementioned patient in whom eosinophilia of 84% was also detected [69].

Fever was described in 138 among 153 patients (90.20%) in whom temperature was analysed. In the great majority it was just referred to as simply presenting fever. In the only 10 patients in whom the exact temperature was described, the mean was 39 °C (± 0.62 °C). In the article in which a higher number (69) of patients was recorded in whom temperature analysis was performed, 58 (84.06%) had fever [41].

Abdominal pain, noted in 200 patients, appears to be the most frequent finding. Unfortunately, it appears not always adequately described. Thus, in most instances only terms as "diffuse", "right hypocondrial pain", "epigastrial", or similar are mentioned.

Weight loss due to the disease was found in 76 of 91 patients (83.52%) in whom a weight control follow-up was made. In the majority of cases only presence or absence of weight loss is stated. It was quantified in only 8 patients, with a range from 4 to 21 kg lost (mean 13.00 ± 5.48 kg). In two articles, the weight loss was classified according to its degree [41,49]: in 20 patients it was slight, in 18 moderate, and in 29 intense or very intense.

Anorexia was described in 53 out of 74 patients (71.6%) in whom this symptom was evaluated in a study [41]. In the literature analysed, anorexia appeared described only in one other patient [69]. Asthenia was described in only 86 patients in Argentina, whereas weakness is commonly associated with mild to moderate anaemia during the acute phase elsewhere [2]. Urticaria was only found in 62 Argentinian patients, although this symptom is considered a distinctive feature in the early stage of fluke invasion [2]. Other less diagnosed symptoms in the country include nauseas in 38 patients, ictericia 23, lithiasis 17, vomiting 11, headache 9, diarrhoea 8, and constipation 6.

#### Co-infection with other parasites

Co-infections of fascioliasis with other protozoan and helminth species have recenly proved to be the usual rule in human fascioliasis endemic areas [3,19-22,44]. Clinical synergistic associations of fascioliasis with other pathogens are believed to be at the base of high morbidity and mortality rates in children [66], immunological responses being markedly suppressed and concomitant infection being exacerbated following liver fluke infection [12,96].

In Argentina, unfortunately patient analyses do not appear to have particularly focused on co-infections. The most frequently reported parasite co-infecting with *F. hepatica *appears to be *Echinococcus granulosus*, including a total of 14 patients reported to simultaneously present both parasites. All of these cases were from an hydatidosis endemic zone in Mendoza province [87,97]. This relative high number of fascioliasis-hydatidosis co-infected patients is outstanding, as such a co-infection has never been detected in human endemic areas of other countries so far.

Other parasite species found in the coprological diagnosis of fascioliasis patients were: *Entamoeba coli *(6 patients), *Giardia intestinalis *(5), *Blastocystis hominis *(4) and *Entamoeba histolytica *(2) among protozoans, and *Enterobius vermicularis *(1*), Strongyloides stercolaris *(1) and *Hymenolepis nana *(1) among helminths. Only two patients have been described to present more than one parasite species additional to *F. hepatica*, namely *G. intestinalis *and *E. vermicularis *in one case [98], and *E. coli *and *G. intestinalis *in another patient [72]. In total, only 30 patients had co-infections with other parasites, of whom only 16 had intestinal parasites. This low amount of co-infection reports may perhaps partly be explained by the geographical origin of the fascioliasis patients, usually dry regions where other helminth diseases are relatively difficult to find, and also by the age of the patients, since most were adult subjects in whom intestinal parasitic diseases are not so prevalent.

#### Treatment

This was specified in 212 patients. Drugs mentioned to have been used were emetine (in 186 patients), triclabendazole (21), cloroquine (4), praziquantel (1), albendazole (1) and male fern extract (1). In two patients, more than one drug was used. A historical analysis shows that emetine was the only drug used up to the end of the 80's, except for the sporadic use of chloroquine [41]. From the beginning of the 90's, triclabendazole became the drug of choice, although praziquantel, albendazole and again emetine were applied in given cases (Table 1).

The first successful treatment report was with emetine intravenous administration in a 38-year-old female patient. Fever and abdominal pain subsided shortly after treatment and coprological analyses showed the disappearance of eggs [33]. Emetine was also sucessfully used a few years later [35] and in another patient to solve the lack of effectiveness of male fern extract [76]. In Argentina, only a few treatment failures with emetine have been reported [39]. An efficacy of 92.4% was obtained in 65 patients in whom *F. hepatica *eggs ceased to be found after treatment [39]. Emetine was even recently used in a family outbreak involving 15 patients, amongst whom two presented severe hypotension. Coprological analyses became negative 60 days after a 10-day-long treatment [47].

Emetine derivatives were the classic drugs and continue to be used today, administered intramuscularly or subcutaneously, at doses of 1-10 mg/kg emetine/day for ten days [8,42,99]. Worldwide, dehydroemetine, at a dose of 1 mg/kg daily for 10-14 days, was considered the therapy of choice a few decades ago [2,8,70]. However, emetine derivatives cause a variety of toxic manifestations involving the heart, liver and digestive tract. A cardiac counter-indication led to the ineffective treatment with chloroquine of four patients to whom emetine could not be prescribed: all of these patients continued to shed fluke eggs after cloroquine administration [41].

Even though there was no triclabendazole formulation for humans available in Argentina, it was, nevertheless, given to patients in its veterinary form, usually with prior consent. The first report of triclabendazole treatment concerned a dose of 10 mg/kg in a 40-year-old male patient with prolonged high temperature and eosinophilia, including a repeated dose 9 weeks later. Clinical symptoms and eggs in stools disappeared thereafter [88].

Another report of triclabendazole use concerned a family outbreak. First, a 26-year-old male patient with fever, eosinophilia, hepatic abscesses, and an ectopic subcutaneous fluke in the abdominal region, was treated with 900 mg/day praziquantel for three consecutive days, with initial disappearance of symptoms. After relapsing two weeks later, the patient was re-treated with 750 mg praziquantel every 8 hours for two days. As symptoms persisted, 10 mg/kg triclabendazole were applied three weeks later. Symptoms disappeared within 48 hours, and normalization of clinical and laboratory parameters was obtained. Four other patients of the same outbreak were also successfully treated with triclabendazole [51]. Triclabendazole was also used at a dose of 400 mg/day for two days in a 20-year-old female [61], as well as with a single 10 mg/kg dose in a 58-year-old woman [85], both with remission of symptoms. In an eight-person family outbreak, six patients received triclabendazole (dosage and protocol not specified) with elimination of egg shedding. Another patient had to receive a second dose to stop egg shedding, and three triclabendazole doses were needed for the last patient [47].

Albendazole at a dose of 400 mg/12 hours was used in a 54-year-old man with remission of clinical manifestations within days. This report stated that albendazole was used for it was impossible to obtain triclabendazole [62]. Although Egaten^® ^(triclabendazole, the drug of choice for human treatment at present) has been recently available from WHO [13,75] it has been still never applied in Argentina. Nitazoxanide has been approved for human use in Argentina for fascioliasis, but no reports have been found in the literature.

### Surgical cases

Even though the number of patients in whom surgery was involved is small (6.9%), publications dealing with surgical cases appear to be proportionally important (15; 26.7%). Surgical description was indeed the main objective of several articles. In the first surgical case reported, actually the third autochthonous case of the country [34], the intervention was described in great detail, but no epidemiological information was provided, not even the province of origin of the patient.

In 45 cases, a surgical procedure contributed to the diagnosis when flukes were unexpectedly found upon liver exploration. The largest number of surgical cases described within one article is 16 [100]. Unfortunately, sometimes no details were given and cases were merely referred to as surgical [72].

In the majority, surgery was indicated due to abdominal pain and biliary obstruction suggestive of lithiasis. Indeed, gallstone disease has recently proved to be one of the effects of advanced chronic fascioliasis, since *F. hepatica *is able to survive up to 9-13.5 years within a human host [9]. Hence, such a high lithiasis proportion suggests that long-term infected patients having been overlooked for a long time has been a relatively frequent situation in Argentina. This agrees with the not unusual long delay in diagnosis already emphasized before, and both observations together pose a question mark about human fascioliasis detection in the country.

In most of these surgical cases with lithiasis suspicion, the patient inhabited a large city (Buenos Aires, Cordoba, Mendoza, Tucuman) as opposed to a rural area where attending a health centre is less usual due to economic reasons or at least complicated due to the long journey that has to be made. This additionally suggests a far greater underestimation of the problem in rural areas.

The importance of intraoperative cholangiography was highlighted in cases in which, even though gallstones were removed, evidence of obstruction observed during the cholangiography led to the finding of flukes [55,89,101]. Gallstones were found and concomitant fascioliasis diagnosis made while performing choledoctomy in another six cases [102]. Two more patients with lithiasis in whom *F. hepatica *was diagnosed upon surgery were described later [42].

In a patient in whom a gallstone was suspected, intraoperative cholangiography showed that in fact, instead of a lithiasis problem, a *F. hepatica *specimen was involved [78]. In another patient operated due to lithiasis suspicion and to resolve a hiatus hernia, stones were neither found at cholecystectomy but flukes were after undertaking an intraoperative cholangiography that indicated estenosis altering normal bile flow to the duodenum [103]. Similarly, no stones were observed but parasites found when performing choledoctomy in another patient diagnosed shortly after [99].

Among four operated patients, fluke infection was detected in two only after a second surgical intervention. In the first operation, cholecistectomy was performed to remove stones but flukes were not detected since intraoperative cholangiography had not been applied. Upon re-operation, fascioliasis was diagnosed when *F. hepatica *was found in the common bile duct [104].

The usefulness of intraoperatory cholangiography for fascioliasis diagnosis was higlighted for cases in which preoperatory diagnosis was difficult [90]. This was concluded when operating five cases due to severe abdominal pain and lithiasis suspicion in only three of them.

In an interesting case, diagnosis was made after surgery to the brain when an expansive parasagital process was diagnosed by means of a carotid arteriogram performed in a patient with memory loss, nominal aphasia and discrete right facial paresia [95]. Two cysts containing *F. hepatica *eggs were extracted from the cortex. The patient died 24 hours after surgery. This is one of the few fatal cases known to be due to fascioliasis worldwide [2]. In Argentina, the other reported fatal case was the first ever to be diagnosed in the country [31], but since it was an imported case it is not accounted for in the present study.

### Present situation and future perspectives

All aforementioned aspects suggest that, in Argentina, human fascioliasis may have been overlooked in the past and its real epidemiological situation may be underestimated in the present, mainly in high risk rural high altitude areas. The recent detection of lymnaeid vector species such as *G. truncatula *[25,26] and *L. neotropica *[15,27], well-known to be linked to high prevalences and intensities of human fascioliasis in neighbouring and close countries such as Bolivia and Peru, add concern to this question. The very high prevalences recently obtained in a survey in Catamarca province [43], of the level of a human hyperendemic situation [1], also point in the same direction.

Thus, the need for appropriate epidemiological studies in the field, in selected areas where lymnaeid vector species of high liver fluke transmission are present, has to be emphasized. Health centres in these areas sould be informed about human infection probabilities, main clinical picture characteristics, adequate diagnosis techniques and the need for Egaten^® ^(triclabendazole for human use) availability. Triclabendazole resistance, recently detected in Argentinian cattle in the province of Neuquen [105], where human infection has already been reported twice [61,62], and its capacity to spread to other areas of the country poses a serious question mark on human treatment in Argentina in the future.

The results of this retrospective overview provide a valuable baseline on which to design adequate multidisciplinary studies on fascioliasis in humans, animal reservoirs and lymnaeid vectors to assess up to which level and in which areas of this very large and environmentally heterogeneous country, human fascioliasis may represent a public health problem in Argentina.

## Methods

### Information sources and review methods

Sources of the literature reviewed include databases, national and multinational web-entries and free collections, multititle packages or web platforms, libraries, and personal e-mail requests when appropriate. Different key words were used when searching in digital sources. Due to the fact that most of the references originate from local publications, the majority of them are not to be found in electronic databases. Special efforts were made to obtain old references published in local journals or very secondary, non-digitalised journals, unpublished reports, abstracts of meetings, symposia, congresses or similar (usually produced by simple photocopying and in very reduced number of copies), and Master's and PhD theses.

The scope was in need to be widened to non-medical journals, as this disease in humans was so neglected in the past that obtaining acceptance for an article dealing with human reports in a medical journal was sometimes difficult. Articles including a high number of human reports but published in veterinary journals as that of Pizzi et al. [106] are good examples.

Human fascioliasis case reports were obtained from the following sources: a) local medical and veterinary articles published in Argentina: 41 references; b) local medical and biological publications from Uruguay: 2 references [35,52]; c) a publication in a medical journal from Spain: 1 reference [36]; d) a publication in an international journal: 1 reference [94]; e) scientific communications at medical and veterinary congresses and meetings (abstract books): 7 references; f) a parasitology book published in Argentina: 1 reference [107]; and g) doctoral theses made at Argentinan universities: 2 references [41,69]. More than half of the references were more than 40 years old.

Great care was taken not to repeat any case, since in many instances duplications could be ascertained and the same patient accounted for in successive publications. Additionally, most of the old articles were published in local, non-peer-reviewed journals, and several were made by non-specialists in fascioliasis. Consequently, data were in many cases only considered at informative or suggestive level. However, information furnished by old published reports proved to be very useful to assess areas where fascioliasis transmission may follow characteristics enabling human infection. In many of these endemic areas, the absence of additional human reports or the very low number of patients diagnosed may be due to inhabitants not attending health centres for different reasons, misdiagnosis of other patients, and/or lack of appropriate surveys.

The problem of being a neglected, usually non-fatal, clinically mild, and non-reportable infection explains why many human cases are never published or reported to national health authorities, nor reported anywhere else. Liver fluke-infected patients described in university theses and afterwards never published anywhere else clearly show this problem.

### Climatic data

For the analysis of the seasonal and annual distributions of human cases with regard to climatic characteristics, only Cordoba province was selected due to its relatively high number of human reports. No other province presented a sufficiently large enough number of cases as to allow for a significant comparative analysis.

Mean monthly data of maximum and minimum temperatures, precipitation and humidity was obtained from the Servicio Nacional de Meteorologia, Buenos Aires. Data analysed only concerned the 1960's and 1970's, when the patients were infected. The aforementioned climatic variables were furnished by ten different meteorological stations throughout the province of Cordoba, strategically selected according to the completeness of the data per station and the appropriate coverage of the geographical distribution of human cases. Geographical coordenates of the ten stations are as follows: Cordoba Aero (31° 19' S, 64° 13' W); Cordoba Observatorio (31° 24' S, 64° 11' W); Dique Cruz del Eje (30° 45' S, 64° 45' W); Dique La Viña (31° 53' S, 65° 02' W); Dique Pisco Huasi (30° 20' S, 64° 00' W); Embalse (32° 11' S, 64° 23' W); Huerta Grande (31° 05' S, 64° 29' W); Rio Cuarto Aero (33° 05' S, 64° 16' W); Rio Tercero (32° 10' S, 64° 08' W); and Villa Dolores Aero (31° 57' S, 65° 08 W).

To investigate the correlation between the moment of diagnosis of the human cases in Cordoba province between the years 1961 and 1981, and different climatic parameters (precipitation, relative humidity, maximum temperature, and minimum temperature), Pearson's correlation coefficient (r) was used by means of Infostat 2008 software. The human cases where correlated with the climatic parameters on a month per month basis and considering time lags of 1, 2, 3, 4, and 5 months considering the incubation period of fascioliasis which can be of up to two to four months. A correlation analysis was also applied to assess the potential relationship between annual precipitation and the number of cases per year in the aforementioned years.

## Competing interests

The authors declare that they have no competing interests.

## Authors' contributions

RMS performed the bibliographical search in the country, analysed the data and contributed to article drafting; VHA performed the bibliographical search on the internet and contributed to article preparation; PC performed the climatic data search and analyses; SMC conceived and designed the study and wrote the paper. All authors read and approved the final manuscript.
